# Cerebral Venous Thromboses in a Patient With No Reported Risk Factors: A Case Report

**DOI:** 10.7759/cureus.35860

**Published:** 2023-03-07

**Authors:** Anjali Patel, Ana Paraiso, Jay P Patel, Rajul Parikh

**Affiliations:** 1 Internal Medicine, HCA Florida Orange Park Hospital, Jacksonville, USA; 2 Internal Medicine, Edward Via College of Osteopathic Medicine, Auburn, USA; 3 Neurology, HCA Florida Memorial Hospital, Jacksonville, USA

**Keywords:** venous thrombosis, cerebral venous thrombosis, vaccine, covid-19, cerebral venous sinus, neurological injuries, rare, women, stroke, cerebral venous sinus thromboses

## Abstract

Stroke is a neurologic condition caused either by brain ischemia or brain hemorrhage, where most cases are a result of ischemic brain injury. Stroke more commonly affects the arterial blood supply of the brain, but in rare cases, it is evoked by the occlusion of the venous sinuses that drain blood from the brain. This phenomenon is known as cerebral venous sinus thrombosis (CVST), also referred to as cerebral sinovenous thrombosis. The pathogenesis of CVST is not completely understood, although common risk factors associated with the condition include obesity, hypercoagulable states, oral contraceptive use, intracranial infections, trauma, and, more recently, coronavirus disease 2019 (COVID-19). Immediate medical intervention is required because CVST can result in increased intracranial pressure and diffuse cerebral edema, which can bring about fatal complications that can lead to early death. However, CVST is challenging to diagnose, as its clinical presentation is highly variable. It can range from headaches to signs of elevated intracranial pressure, including nausea, vomiting, and vision problems.

In this case report, the patient is a 25-year-old previously healthy African American female who presented with a weeklong headache and acute onset of delirium an hour prior to arrival at the hospital. The patient had prior emergency department (ED) visits from different facilities where head imaging was performed and showed negative results allowing her to return home. The patient was then brought by a friend to our ED due to altered mental status and agitation. Initial computed tomography of the head did not reveal acute abnormalities; however, magnetic resonance angiography and magnetic resonance venography revealed evidence of venous sinus thrombosis and lack of flow requiring urgent attention. The patient was then referred to endovascular neurology, but despite medical intervention, the patient’s medical status deteriorated, and she was declared brain dead.

Although rare, this case report emphasizes the atypical presentation and the severity of CVST where a young individual with no significant past medical history presented with neurological symptoms that rapidly progressed to complications that caused her early death.

## Introduction

Stroke is classified into two different types, namely, brain ischemia and brain hemorrhage, with approximately 80% being ischemic cerebral infarction [1]. A large number of ischemic strokes are a result of reduced arterial blood supply to the brain. Another cause that is less common and more difficult to diagnose is obstruction of veins that drain blood [1]. Cerebral venous sinus thrombosis (CVST) is a condition in which there is blood clot formation in the venous sinuses of the brain. Blood clot formation prevents the proper draining of blood from the brain, resulting in the damage of vessels and the leaking of blood into the surrounding brain tissues, leading to a hemorrhage [2].

The more common stroke symptoms include sudden numbness or weakness of extremities, drooping of the face, and trouble speaking [3]. Symptoms of CVST are associated with increased intracranial pressure, including focal neurologic deficits [4]. Approximately 90% of individuals with a CVST attack will present with a headache, along with other more or less common symptoms, including seizures, nausea, vomiting, difficulty speaking or understanding language, blurred vision, or weakness in one, two, or all four extremities [5].

Urgent neuroimaging is necessary for the diagnosis of CVST. When CVST is suspected, it is recommended to obtain brain magnetic resonance imaging (MRI) and magnetic resonance venography (MRV) or brain CT with CT venography, which will show an absence of flow in a venous sinus [6]. The most sensitive imaging method is T2-weighted gradient echo and T2 susceptibility-weighted MRI sequences, which will exhibit a hypointense area indicating acute thrombus [7,8]. In addition to neuroimaging, laboratory tests can be measured; however, there is no confirmatory laboratory test that can indicate CVST. Due to this, it is suggested to obtain a complete blood count, chemistry panel, prothrombin time, and activated partial thromboplastin time, which will demonstrate possible etiologies of CVST such as infection, inflammatory condition, and hypercoagulability [6].

Due to the severity of the condition, diagnosis and treatment of CVST is a time-sensitive issue due to the development of early deterioration and eventual death. A study found that the median time was 13 days between symptom onset and death and five days between diagnosis and death [8]. Moreover, approximately 5% of patients with CVST die during the acute phase of the disease due to transtentorial herniation from multiple hemorrhagic lesions, focal mass effect, and diffuse edema [8]. This indicates the severity of the condition and the rapid development of complications caused by CVST. The most common complications of acute CVST are venous infarction and subarachnoid hemorrhage, which are both caused by the increased venous pressure that leads to the rupture of dilated venous structures [9]. Another less common complication is pulmonary embolism, which suggests dislodgement of the thrombosis usually from the sagittal sinus [9]. Additionally, the risk of developing recurrent CVST and other thrombotic events is found to be approximately 2-4% [6]. Risk factors include a history of prior venous thromboembolism, thrombophilia, male sex, polycythemia/thrombocythemia, and Black race [6].

## Case presentation

A 25-year-old African American female patient was brought to the emergency department (ED) with an acute onset of headache and delirium that began an hour prior to arrival at the ED. She had been experiencing headaches for about a week before she came to our ED. She had visited outside facilities in the area during that time where she was given the analgesic combination of acetaminophen-butalbital-caffeine and intramuscular ketorolac. Three days prior to the presentation, she went to an outside ED and had a non-contrast head CT performed, which was interpreted as showing no significant findings, allowing for the patient to be discharged. Two days prior to the presentation, she went to an urgent care facility where she was treated conservatively.

A day prior to the presentation, the patient reported the continuation of the initial headache, but now with pain down the left side of the neck with associated nausea. The patient presented to a different ED where head imaging also showed negative results. At this visit, the patient was told that her symptoms were related to possible migraine headaches for which she was provided an analgesic and a muscle relaxant prior to discharge.

The patient was brought to our ED by a friend who stated that she became altered, agitated, and nonverbal en route. Upon presentation to our ED, the patient was severely confused and agitated but was still able to follow simple commands, phonate/moan in response to being conversated with, and had a spontaneous movement of all extremities. Initial vital signs demonstrated patient was hypertensive (Table 1). Pertinent physical examination findings included acute alteration of mental status where she was found to be alert and oriented x0. Due to the patient's emergent condition, cranial nerves were not examined. However, on the Glasgow Coma Scale (GCS) examination, the patient had spontaneous eye opening, incomprehensible sounds on verbal response, and localizing to pain on the motor response for a total score of 11.

**Table 1:** Initial vital signs

Vital sign	Value	Normal range
Temperature (F)	98	97.8 to 99.1
Blood pressure	142/85	120/80
Pulse	95	60 to 100
Respiratory rate	20	12 to 20 BPM
Oxygen saturation	98	95 to 100
BMI	39.7	18.5 to 24.9

**Figure 1:**
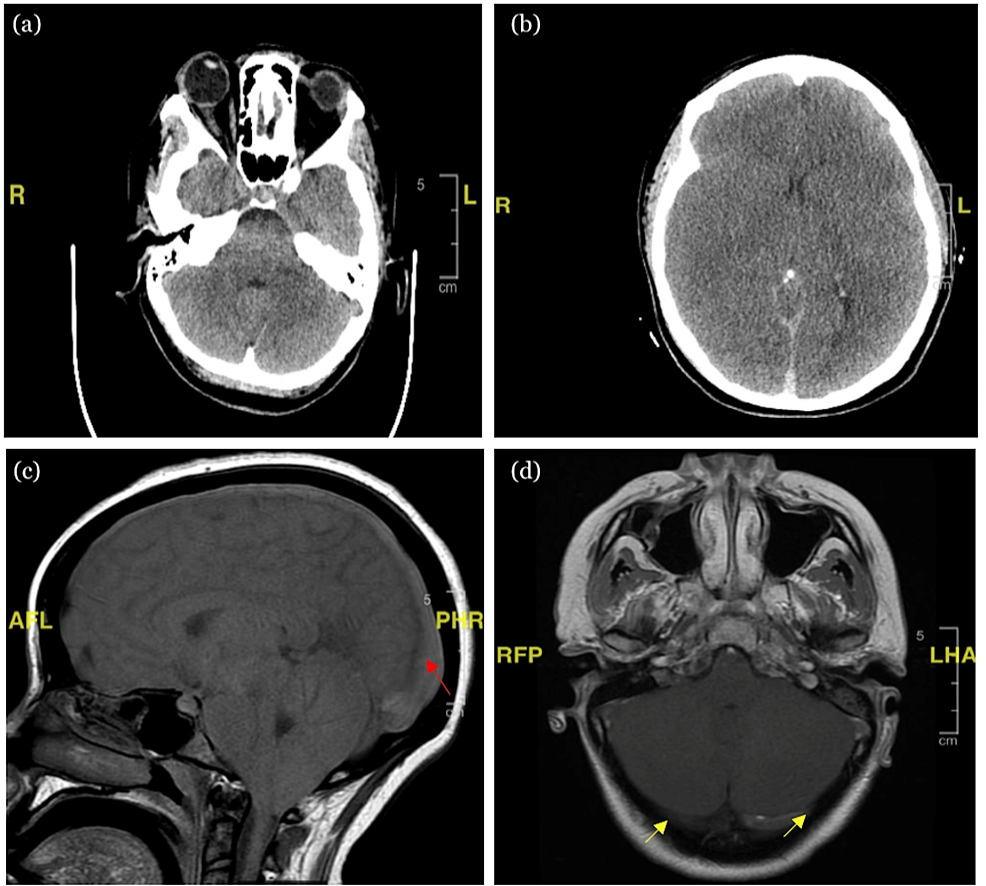
Imaging of the brain (A) Initial non-contrast-enhanced CT of the brain with no acute findings. (B) CT of the brain venography demonstrating diffuse cerebral edema. MRI of the brain (C) sagittal view (red arrow) and (D) axial view (yellow arrows) showing evidence of sinus thromboses and lack of blood flow.

CT of the brain was performed 30 minutes following arrival, which reported no acute findings (Figure 1A). Initial laboratory findings demonstrated microcytic anemia and leukocytosis (Table 2). About 26 minutes following the CT scan, she was found to have seizure-like activity with muscle twitching in all four extremities so she was given 1 mg lorazepam. She then experienced what appeared to be decerebrate posturing and acute decline in her GCS where she became completely unresponsive, nonverbal, and not following any commands. Thirty minutes later, she was intubated for airway preservation after being noted for decerebrate posturing with acute decline in neurological status. Following the finding of fixed and dilated pupils on repeat physical examination, on-call neurology was consulted. While awaiting further imaging and studies, the patient was admitted to the intensive care unit for further investigation. Concurrently, the patient had been started on IV hypertonic saline, IV mannitol, and IV lorazepam, secondary to differential diagnosis, including cerebrovascular accident, drug overdose, encephalitis, epidural hematoma, seizure disorder, and subarachnoid hemorrhage. Four hours after intubation, on neurological exam, the patient was not responsive to sternal rub or painful stimuli with nail bed pressure and had absent gag, corneal, and cough reflexes. The patient did have minimal delayed toe movement in response to plantar stimulation although no clear withdrawal was noted.

**Table 2:** Initial laboratory studies PTT: partial thromboplastin time; PT: prothrombin time; INR: international normalized ratio; CMP: comprehensive metabolic panel; CBC: complete blood count; HGB: hemoglobin; HCT: hematocrit; MCV: mean corpuscular volume; MCH: mean corpuscular hemoglobin; MCHC: mean corpuscular hemoglobin concentration; RDW: red cell distribution width; PLT: platelet; B-HCG: beta-human chorionic gonadotropin.

Laboratory study	Value	Normal range
Coagulation studies		
PTT	22 sec	22.9-35.2 sec
PT	10.3 sec	9.5-12.1 sec
INR	1	0.9-1.1
CMP		
Na	141	136-145 mMol/L
K	3.1	3.8-5.0 mMol/L
Cl	104	99-110 mMol/L
CO2	25	21-32 mMol/L
Glucose	121	74-106 mg/dL
CBC		
WBC	14.8	3.4-10.7 K/mcl
RBC	3.72	4.00-5.40 M/mcl
HGB	7.9	12.0-16.0 G/dl
HCT	25.9	38-47%
MCV	69.6	81-100 fL
MCH	21.2	27-35 pg
MCHC	30.5	33-36 g/dL
RDW	17.6	11.0-13.5%
PLT	251	125-375 K/mcL
Troponin	5.9	4-60.30 pg/ml
B-HCG	Negative	

The patient had several imaging and laboratory studies conducted starting with a repeat head CT, followed by urinalysis, blood cultures, toxicology screen, CT angiogram (CTA) of the neck, CT venogram of the brain, electroencephalogram (EEG), and both MRI and MRV of the brain. The repeated head CT scan demonstrated massive diffuse cerebral edema with the absence of cortical sulci, cerebrospinal fluid (CSF) spaces, and basal cisterns, progression of upper and downward cerebellar and uncal herniation, and possible pseudo-subarachnoid sign. Urinalysis and blood cultures were both unremarkable. The toxicology screen was also unremarkable except for a positive barbiturate level likely due to the patient's analgesic prescription. Impression of the CTA of the neck was reported to show very poor opacification of all intracranial arteries, highly likely representing severely increased intracranial pressures. The CT brain venogram demonstrated diffuse cerebral swelling with tonsillar herniation and effacement of the basal cisterns and suspicion of bilateral transverse sinus occlusion (Figure 1B). MRI of the brain showed evidence of sinus thrombosis, including the confluence of sinuses, bilateral transverse sinuses, bilateral sigmoid and jugular, probable thrombus in the straight sinus, mass effect with lack of cortical sulci, and cerebellar tonsillar herniation with cerebellar edema (Figures 1C, 1D). MRV demonstrated a generalized lack of flow-related enhancement throughout the jugular veins and the transverse sinus, sigmoid confluence of sinuses, straight sinus, and intracerebral veins. Lastly, the patient’s EEG study was interpreted as electrocerebral silence.

Due to MRV brain findings demonstrating nearly a total lack of flow of both deep and superficial venous systems, indicating extensive venous occlusion, the diagnosis of CVST was made. Transfer to another hospital for possible further intervention of the sinus venous thrombosis by an endovascular neurology specialist was advised. Upon arrival, the neuro-interventional team placed an external ventricular drain (EVD) without any successful CSF return. A subsequent cerebral arteriogram was performed, which demonstrated no intracranial blood flow.

Ultimately, the patient’s medical presentation had drastically progressed before any interventional steps could be taken. The patient met the criteria for brain death due to the absence of all reflexes, hemodynamic instability, hypothermic, hypernatremic with central diabetes insipidus, and absent cerebral blood flow; therefore, 37 hours after the presentation, the patient was pronounced deceased.

## Discussion

The term “stroke” encompasses numerous cerebrovascular diseases. This is one of the common causes of long-term disability and even death in many cases [10]. Data reported from the American Heart Association (AHA) statistical update demonstrated that in the years 2015-2018, an estimated 7.6 million Americans ≥ 20 years of age self-reported an incidence of stroke, a prevalence estimated at 2.7% [11]. Moreover, about 795,000 individuals experience a new or recurrent stroke annually, with 610,000 of these events being the first episode [11].

According to the analysis of the data from the Global Burden of Disease (GBD) study discussed in the AHA statistical update, 87% of stroke risk is tied to common modifiable risk factors such as hypertension, hyperglycemia, hyperlipidemia, obesity, renal dysfunction, and behavioral practices such as smoking, unhealthy diet, and a sedentary lifestyle [11]. In addition, social determinants of health are also found to have a major impact on the development of stroke. Adverse work conditions, long work hours, and smaller social networks with a lack of support through contact with family members, friends, and neighbors are also very important to consider as links to higher stroke risk [11].

CVST is a rare form of stroke, only affecting about five in a million people per year [12]. Although rare, it is a serious disorder with increased risks of disability and death [12]. The risk of a CVST is also greatly seen in newborn infants in their first month of life [2]. Moreover, overall rates of CVST are higher in women with a 3:1 ratio of cases in women compared to men [13,14]. Data from a prospective observational study designed to investigate the gender-specific manifestations of CVST demonstrated 75% of the CVST patients were women and a gender-specific risk factor, such as oral contraceptives, pregnancy, or hormonal replacement therapy, was present in 65% of these women [13].

With CVST being a form of stroke, the previously mentioned risk factors and attributable causes should be considered along with others. Although the risk for CVST is seen greatly in infants, it can affect individuals of all ages and has varying risk factors per age group. Some specific risk factors to consider in the children and infants population include blood clotting disorders, sickle cell anemia, chronic hemolytic anemia, infections, dehydration, trauma to the head, maternal infections, and heart diseases, both congenital and acquired [2]. In the adult population, risk factors vary slightly and should include, but not be limited to, conditions with abnormal blood clotting, various types of cancers, collagen vascular diseases, obesity, both variants of inflammatory bowel disease, genetic predispositions, oral contraceptives, and the full course of a woman’s pregnancy [5].

In addition to the risk factors previously mentioned, more recent studies found an association between CVST occurrence and coronavirus disease 2019 (COVID-19). A case study reported CVST in a previously healthy 29-year-old female due to the hypercoagulability effects of COVID-19 [15]. This indicates that although more serious complications from COVID-19 are associated with older populations with comorbidities, it can also be seen in young populations as an atypical presentation such as CVST. However, our patient tested negative for COVID-19 upon admission, narrowing down our search for the etiology by one possible cause.

Our patient had an atypical presentation without any significant risk factors, including medical history or medications. However, with the quick onset of the patient’s presentation, a thorough history was not obtained, leaving a lot of questions unanswered. The patient was not capable at the point of admission to provide a personal medical history, leaving us with limited information to provide a definite reason for the patient’s outcome. With there being many factors and comorbidities to consider as the ultimate cause of the patient’s outcome, a few to highly consider based on the patient’s profile include oral contraceptives and sickle cell anemia. Oral contraceptives are widely used and with the patient being female, this cannot be ruled out. In a meta-analysis study, it was found that women of reproductive age who were taking oral contraceptives are approximately sevenfold more likely to develop CVST compared to women who were not taking it [16]. This may be due to the thrombotic biological side effects of the use of oral contraceptives.

Furthermore, upon discussion with the patient’s mother, some family history of sickle cell anemia was gathered, leaving us to question if it was a component of the patient’s medical history as well. Sickle cell anemia is a hypercoagulable state, which leads to the formation of microvascular thrombosis. Some hypotheses exist as to why this is the case, including erythrocyte adhesion, endothelial dysfunction, leukocyte activation in the setting of chronic inflammation, platelet aggregation, coagulation defects, and free hemoglobin-induced oxidative damage [17]. A cross-sectional study demonstrated that a history of venous thromboembolism was found in 25% of adult individuals with sickle cell disease [17].

## Conclusions

CVST is a medical complication associated with many factors, more commonly including clotting disorders, illnesses, and hypercoagulable states such as pregnancy. Although there is no pronounced answer for the ultimate declaration of the patient’s brain death, her case allowed for the understanding of the vast range of possible causes of CVST and the time-sensitive need for interventions, once diagnosed. Early recognition and timely management of CVST is important to prevent serious complications such as motor and sensory deficits, including extremity movement and visual problems, permanent brain damage, and in severe cases such as this one, death.
